# Adjuvant Screen Identifies Synthetic DNA-Encoding Flt3L and CD80 Immunotherapeutics as Candidates for Enhancing Anti-tumor T Cell Responses

**DOI:** 10.3389/fimmu.2020.00327

**Published:** 2020-02-25

**Authors:** Amy Haseley Thorne, Kirsten N. Malo, Ashley J. Wong, Tricia T. Nguyen, Neil Cooch, Charles Reed, Jian Yan, Kate E. Broderick, Trevor R. F. Smith, Emma L. Masteller, Laurent Humeau

**Affiliations:** ^1^Inovio Pharmaceuticals Inc., San Diego, CA, United States; ^2^Inovio Pharmaceuticals Inc., Plymouth, PA, United States

**Keywords:** Flt3L, CD80, vaccine, immune, cancer

## Abstract

Overcoming tolerance to tumor-associated antigens remains a hurdle for cancer vaccine-based immunotherapy. A strategy to enhance the anti-tumor immune response is the inclusion of adjuvants to cancer vaccine protocols. In this report, we generated and systematically screened over twenty gene-based molecular adjuvants composed of cytokines, chemokines, and T cell co-stimulators for the ability to increase anti-tumor antigen T cell immunity. We identified several robust adjuvants whose addition to vaccine formulations resulted in enhanced T cell responses targeting the cancer antigens STEAP1 and TERT. We further characterized direct T cell stimulation through CD80-Fc and indirect T cell targeting via the dendritic cell activator Flt3L-Fc. Mechanistically, intramuscular delivery of Flt3L-Fc into mice was associated with a significant increase in infiltration of dendritic cells at the site of administration and trafficking of activated dendritic cells to the draining lymph node. Gene expression analysis of the muscle tissue confirmed a significant up-regulation in genes associated with dendritic cell signaling. Addition of CD80-Fc to STEAP1 vaccine formulation mimicked the engagement provided by DCs and increased T cell responses to STEAP1 by 8-fold, significantly increasing the frequency of antigen-specific cells expressing IFNγ, TNFα, and CD107a for both CD8^+^ and CD4^+^ T cells. CD80-Fc enhanced T cell responses to multiple tumor-associated antigens including Survivin and HPV, indicating its potential as a universal adjuvant for cancer vaccines. Together, the results of our study highlight the adjuvanting effect of T cell engagement either directly, CD80-Fc, or indirectly, Flt3L-Fc, for cancer vaccines.

## Introduction

Much progress has been made in the field of immuno-oncology in recent years, revealing the promise of harnessing an individual's immune system to effectively target cancer cells. An effective immune response requires a coordination of several working parts: tumor-associated antigens must be processed and presented to antigen-specific T cells, the T cells must be activated and expanded, and then traffic to and accumulate at the tumor site, maintaining activity long enough to effectively destroy a tumor in an immuno-suppressive microenvironment (1). Although immune checkpoint therapy has made some of the greatest strides in this field to date (2), therapy-associated toxicities can be prohibitive and not all tumors respond (3, 4). Importantly, studies with the dendritic cell vaccine Sipuleucel-T (Provenge®) have shown that using a vaccination strategy to target cancer is a viable therapeutic option. However, this therapy has shown only modest efficacy, is cost-prohibitive, and requires extensive *ex vivo* manipulation: immune cells are isolated from the patient's blood, activated in a laboratory, and then infused back into the patient (5, 6). Plasmid DNA vaccination provides a simple and accessible approach to immune therapy, generating an activated immune response to tumor-associated antigens *in vivo*. Vaccination with highly optimized DNA cancer vaccines and delivered by the CELLECTRA® electroporation (EP) device for cancer has been highly effective in the preclinical space (7–11) and results from a phase 2b clinical trial testing VGX-3100 for patients with cervical intraepithelial neoplasia showed this therapy to be safe, efficacious, and immunogenic (12). It has been well-documented that the specific targeting of immune cells by molecular adjuvants increases the immune response to viral associated antigens (13–16), thus the potential exists to further increase the magnitude of the vaccine-elicited responses against tumor-associated antigens (TAAs) enabling tumor control.

Dendritic cells (DCs) are professional antigen-presenting cells that play a central role in priming T lymphocytes. At the initiation of the immune response, DCs both present antigen and up-regulate co-stimulatory molecules. In response to intramuscular injection and electroporation (IM/EP) of plasmid DNA, DCs migrate to the site of inoculation and present antigen either via cross-presentation or direct transfection (17–19). However, DCs are usually found in small numbers in the muscle and they typically present as functionally immature (20). Thus, enhancing DC activity to prime T cells with optimal efficiency in this setting could improve anti-tumor immune responses. FMS-like tyrosine kinase 3 ligand (Flt3L) is a potent DC specific growth factor that has been shown to expand and mature DCs in both mice and humans (21). Studies have shown that administration of recombinant Flt3L increases the total number of lymphocytes, granulocytes, and monocytes, and massively mobilizes lymphoid/myeloid CD34^+^ progenitor cells into peripheral blood (22, 23). Synergy between Flt3L and DC recruitment for the advantage of immune therapy has been well-documented (24–26). CD80 is an activation molecule on APCs including DCs that interacts with CD28 on T cells enabling T cell proliferation and activation (27). CD80-T cell interaction mechanisms have been shown to maintain a strong T cell activation through both co-stimulation and by blocking inhibition induced by PD-1 and PD-L1 (28). Thus, Flt3L and CD80 represent targets for adjuvant interventions to enhance T cell-mediated anti-tumor immune responses.

In this study, we explored the ability of plasmid-encoded adjuvants to mediate T cell responses to tumor antigens in mice. We screened over twenty synthetic DNA-encoded adjuvants targeting immune processes thought to be associated with anti-tumor responses. We show that formulating the DNA vaccine STEAP1 with Flt3L-Fc mobilized DCs to the site of injection and resulted in a significant increase in the antigen-specific immune response to the target tumor antigen. Several of the candidate adjuvants which enhanced the immune response to the target tumor antigen were involved in direct T cell activation. CD80-Fc provided the most robust antigen-specific immune response to STEAP1 and may be considered a potential universal adjuvant as it significantly boosted several additional cancer vaccines of interest. Together, our data suggests that cancer vaccines may be boosted by both indirectly engaging T cells via influx of DCs and also by directly engaging T cells through co-stimulation and warrants the further investigation of CD80-Fc and Flt3L-Fc as potential “off the shelf” adjuvants to enhance tumor targeting immune responses.

## Results

### Adjuvant Screen Identifies T Cell Co-stimulators and the Dendritic Cell Activator Flt3L as Potential Candidates to Enhance Anti-cancer Vaccines

**Figure 1:**
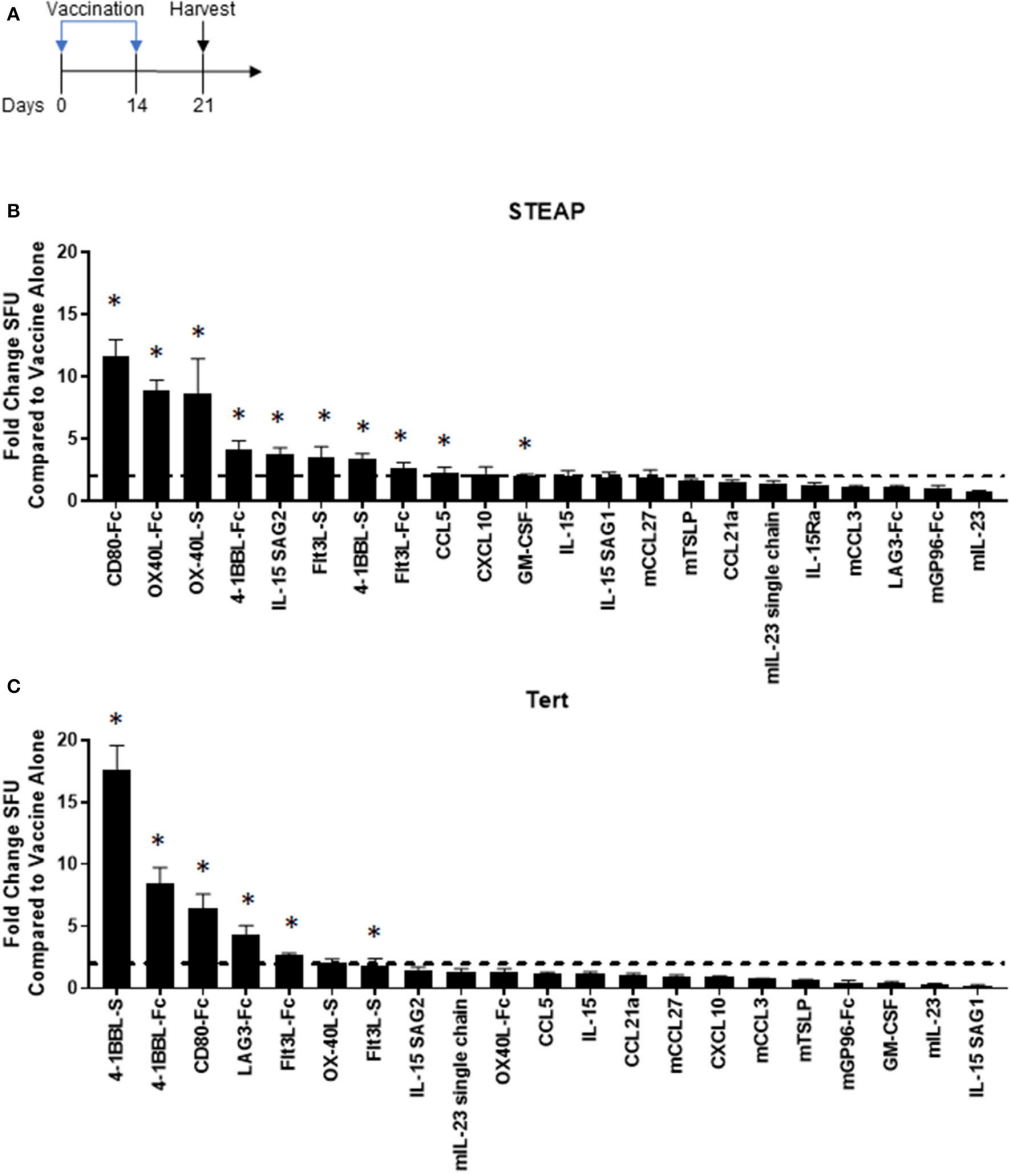
Adjuvant screen identifies T cell co-stimulators and the dendritic cell activator Flt3L as candidates for cancer vaccines. **(A)** Diagram of the experiment. Mice were vaccinated on days 0 and 14 and spleens were harvested on day 21 for analysis. **(B,C)** Immunogenicity fold change of STEAP1 or TERT plus adjuvant compared to STEAP1 or TERT alone by IFNγ ELISpot responses. The fold change boost is defined as an increase in IFNγ ELISpot response in stimulated splenocytes from mice treated with adjuvant plus antigen as compared to mice treated with antigen alone. *N* = 8–10 mice. *p < 0.05.

We designed and tested over twenty plasmid DNA-encoded genetic adjuvant candidates, chosen based on defined biological mechanisms of action (chemokines, cytokines, and ligands for T cell co-stimulation) associated with anti-tumor immune responses. Plasmids were individually formulated with a DNA vaccine targeting a synthetic consensus (SynCon®) antigen of either human Six-Transmembrane Epithelial Antigen of Prostate 1 (STEAP1) or murine Telomerase Reverse Transcriptase (TERT) and administered intramuscularly to mice using the CELLECTRA® 3P device (IM/EP). Antigen-specific immune responses from splenocytes were measured following peptide stimulation by IFNγ ELISpot (Figure 1). Of the 21 adjuvants tested, 10 significantly boosted the immune response to hSTEAP1 tumor antigen by at least 2-fold (Figure 1B), and six boosted the immune response to TERT tumor antigen by at least 2-fold (Figure 1C). Overall, the candidate adjuvants which play a role in direct T cell activation (CD80, OX-40L, and 4-1BB-L) showed the greatest enhancement in antigen-specific T cell responses to vaccination with either STEAP1 or TERT. The T cell co-stimulator CD80 was the strongest immuno-enhancer: increasing STEAP1-specific T cell responses by 11.6 fold and increasing TERT-specific T cell responses by 6.44 fold. Independent to direct T cell engagement, vaccine formulation with Flt3L was associated with enhanced T cell responses to both tumor antigens by >2-fold (STEAP1: 2.67 fold; TERT: 2.7 fold). Therefore, we focused the following studies on CD80 and Flt3L.

### Synthetic DNA-Encoded Murine CD80-Fc and Flt3L-Fc Design and Expression *in vitro* and *in vivo*

**Figure 2:**
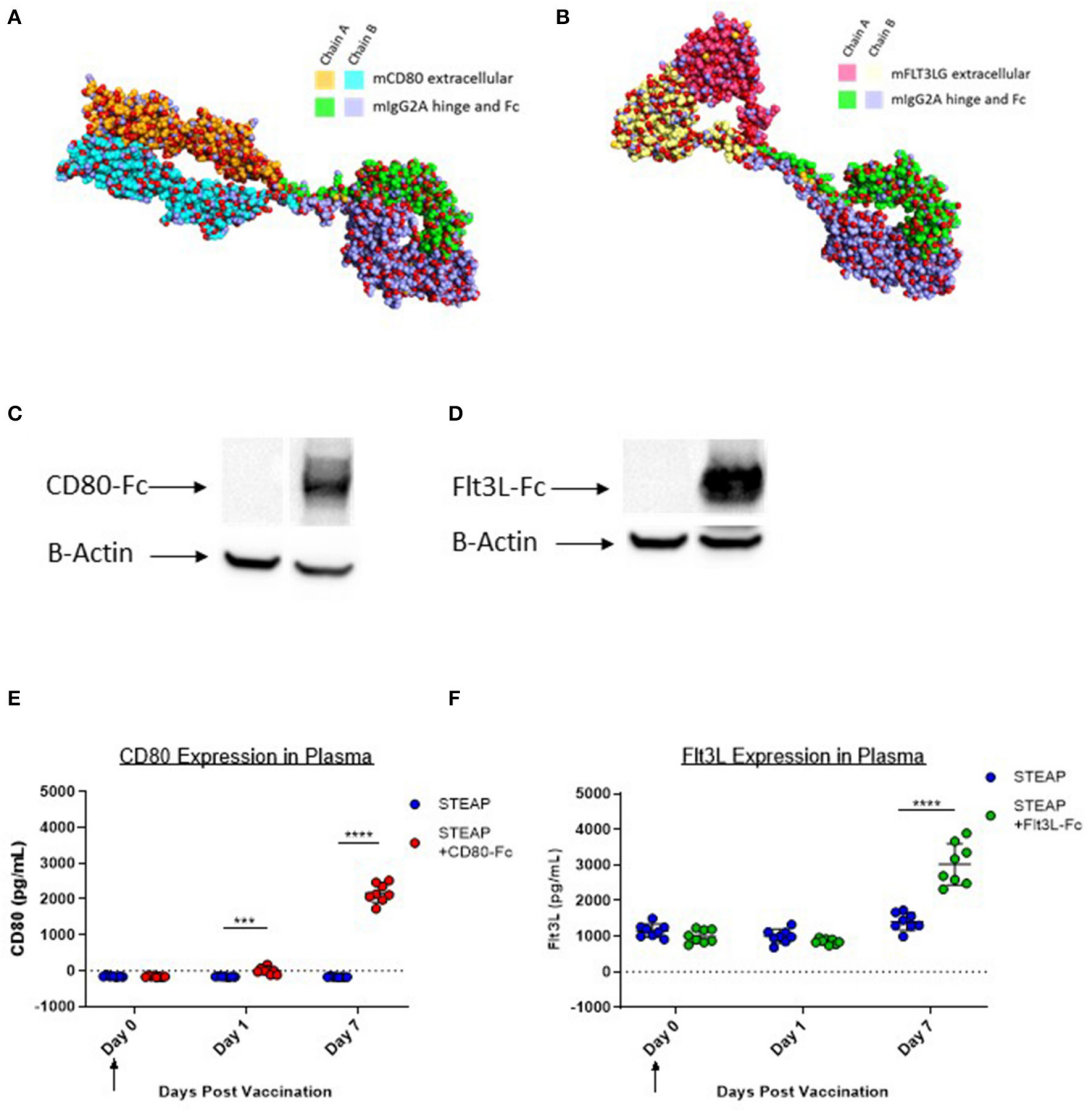
CD80-Fc and Flt3L-Fc express *in vitro* and *in vivo*. **(A)** Comparative model of murine Flt3L extracellular domain-murine IgG2A fusion. **(B)** Comparative model of murine CD80 extracellular domain-murine IgG2A fusion **(C,D)** 293T cells were transfected and Flt3L-Fc **(C)** and CD80-Fc **(D)** expression was analyzed by Western blot and compared to pVAX control. **(E,F)** Mice were immunized and Flt3L-Fc **(E)** and CD80-Fc **(F)** expression was measured in the plasma over time. *N* = 8 mice, ***p < 0.001, ****p < 0.0001.

Murine CD80-Fc and murine Flt3L-Fc were designed based on type I single pass transmembrane proteins with well-defined extracellular, transmembrane, and intracellular domains. Both of these proteins possess native signal peptides which were incorporated into the design. For the Fc fusions, the extracellular regions were harvested and fused with a fully murine IgG2A Fc. We performed comparative modeling of these constructs using the MacroModel and Crosslink Proteins features as implemented in Bioluminate (Schrödinger, LLC, New York, NY, 2019), to better understand the potential implications of putatively unstructured regions between the more well-ordered domains, and also to enable identification of sites for optimization if necessary (Figures 2A,B). To ensure expression of these novel constructs, we first transfected 293T cells with either the construct of interest or the control empty vector plasmid (modified pVAX). Cell lysates were analyzed by western blot. Our results indicate that both CD80-Fc and Flt3L-Fc proteins properly express *in vitro* (Figures 2C,D). To address *in vivo* expression following plasmid DNA administration via IM/EP, we administered formulations of STEAP1 or STEAP1 with adjuvant, and then assayed systemic levels of each protein at days 0, 1, and 7 by ELISA. We show in Figures 2E,F that IM/EP injection of plasmid-DNA encoding CD80-Fc or Flt3L-Fc results in expression of the respective proteins with values of 2,341 and 1,610 pg/ml, respectively, in the plasma of mice 7 days post treatment.

### Flt3L-Fc Significantly Increases Antigen-Specific T Cell Responses to STEAP1 Tumor Antigen

**Figure 3:**
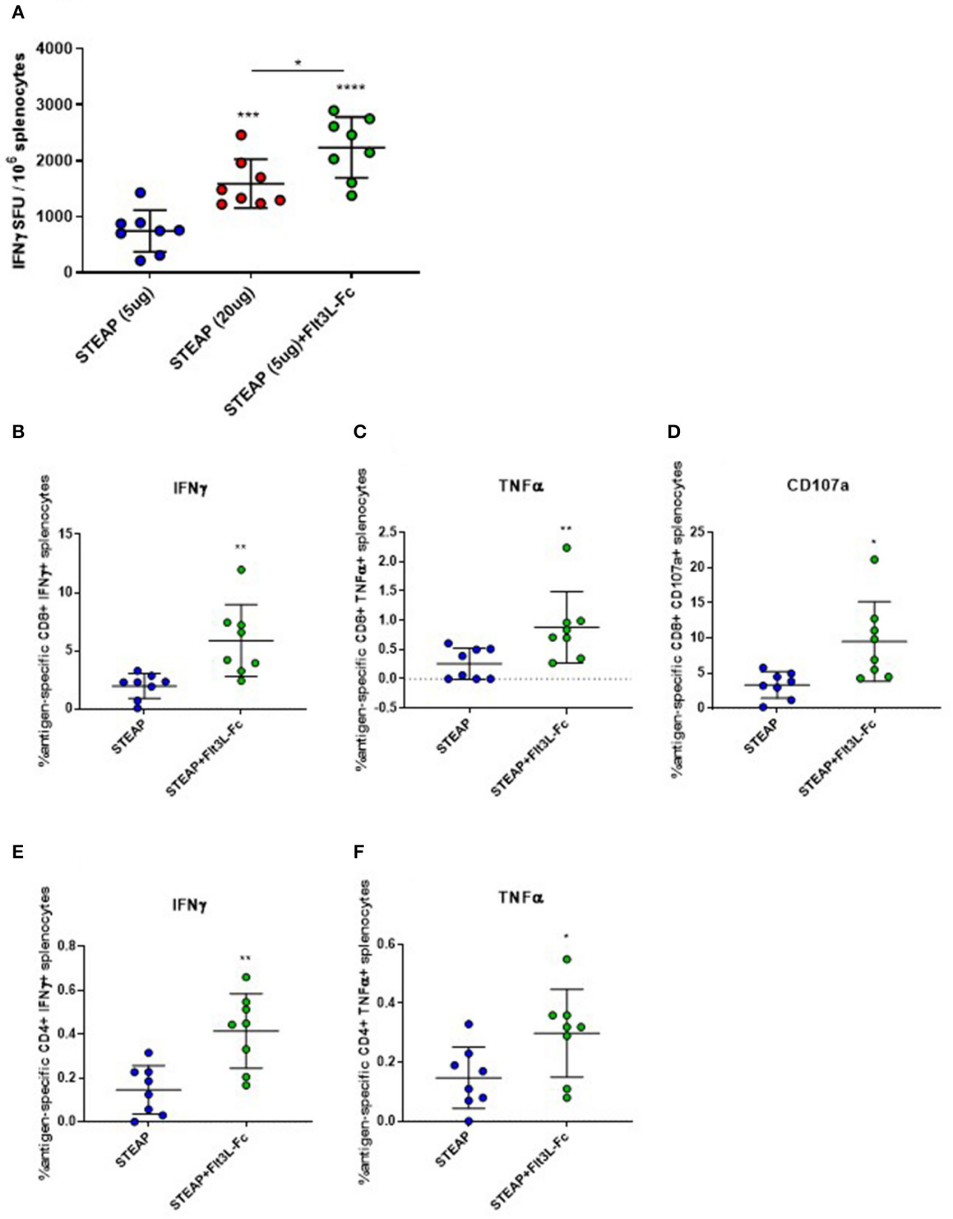
Flt3L-Fc increases antigen specific T cell responses to STEAP1. **(A)** Mice were immunized biweekly according to Figure 1A and an IFNγ ELISpot was run on splenocytes to assess antigen-specific T cell responses to STEAP1. **(B–F)** Intracellular cytokine staining was done on splenocytes to characterize CD8^+^ **(B–D)** and CD4^+^ **(E,F)** functional T cell responses from mice immunized with STEAP1 alone or in combination with Flt3L-Fc. *N* = 8, *p < 0.05, **p < 0.01, ***p < 0.001, ****p < 0.0001.

Our initial adjuvant screen examined one dose level for antigen and adjuvant, next we proceeded to examine the effect of STEAP1 dose range on T cell responses. We compared two different dose levels of STEAP1, 5, and 20 ug, where 5 ug was chosen as a sub-optimal dose for the initial screen to assess adjuvanting, and 20 ug is the dose level which affords maximal T cell response prior to plateau (data not shown). There was a significant increase in STEAP1-specific T cell responses at a 20 ug dose of STEAP1 compared to a 5 ug dose (Figure 3A). The addition of 19 ug Flt3L-Fc to 5 ug of STEAP1 significantly enhanced the antigen-specific T cell response to levels greater than the plateau level afforded by STEAP1 alone at 20 ug, indicating that the addition of Flt3L-Fc to STEAP1 vaccination is not merely dose-sparing.

We proceeded to characterize the effect of Flt3L-Fc by specifically analyzing the T lymphocyte phenotype by flow cytometry. We performed intracellular cytokine staining on peptide-stimulated spleen cells from mice treated with STEAP1 formulated with Flt3L-Fc compared to STEAP1 alone. Results show that both CD8^+^ and CD4^+^ T cell populations from mice treated with STEAP1 formulated with Flt3L-Fc possess a significantly greater frequency of STEAP1-specific cells expressing IFNγ and TNFα compared to mice treated with STEAP1 alone (Figures 3B,C,E,F). The CD8^+^ T cell population also displayed a significantly enhanced frequency of cells expressing the degranulation marker, CD107a, when Flt3L-Fc is formulated with STEAP1 (Figure 3D). In summary, these results indicate that Flt3L-Fc formulated with STEAP1 drives an increase in the quantitative and qualitative functional T cell response.

### Flt3L-Fc Mobilizes and Activates Dendritic Cell Populations

**Figure 4:**
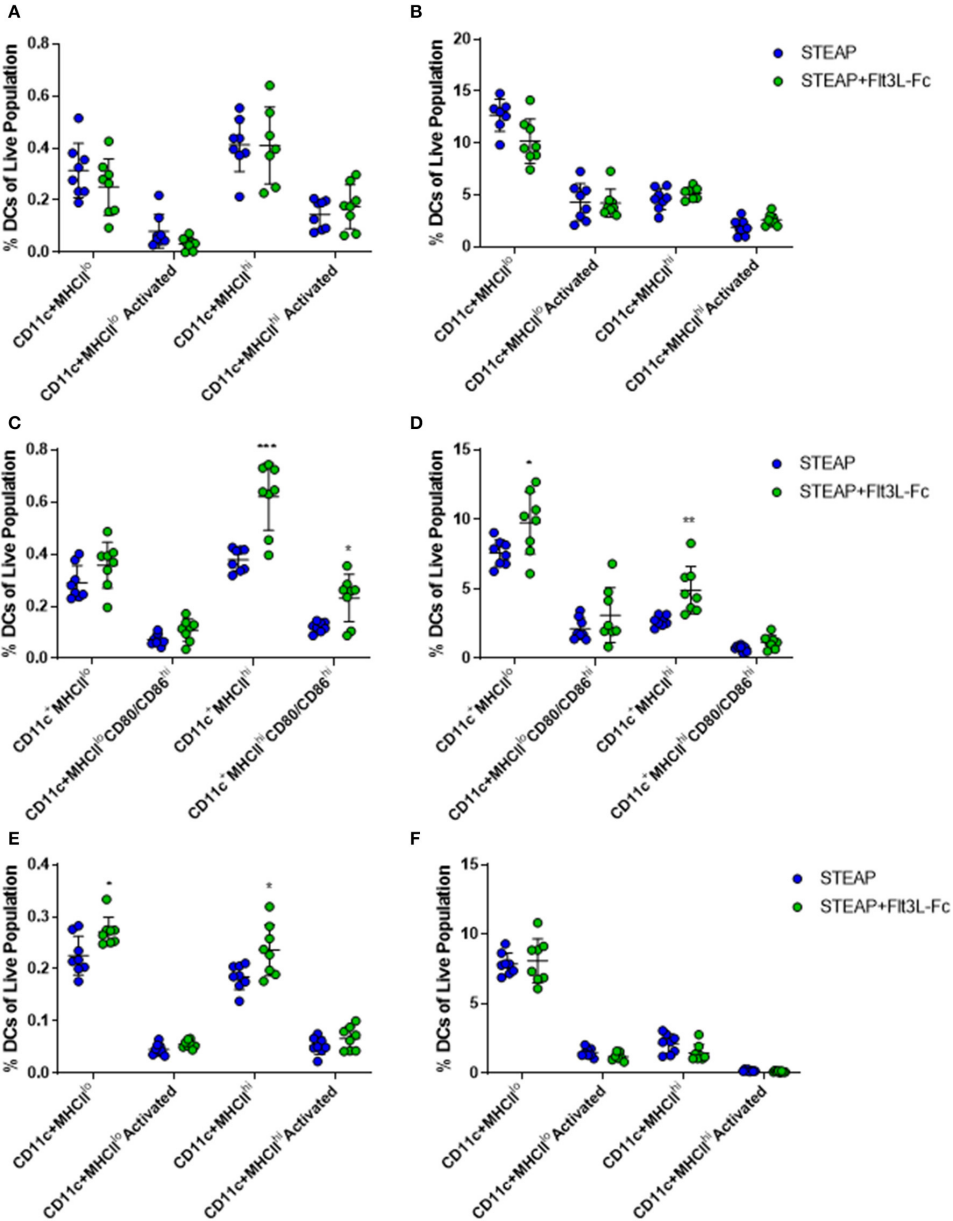
Flt3L-Fc significantly increases dendritic cell migration and trafficking. Mice were immunized weekly and the draining lymph node and TA muscle were harvested 24 h following each treatment. **(A–F)** Flow cytometry was performed on cells harvested from the dLN **(A,C,E)** and TA muscle **(B,D,F)** to assess DC populations 24 h after the first **(A,B)**, second **(C,D)**, and third **(E,F)** doses. *N* = 8, *p < 0.05, **p < 0.01, ***p < 0.001.

To investigate the mechanism of action associated with enhanced T cell responses with the addition of Flt3L-Fc, mice were vaccinated weekly with STEAP1 alone or STEAP1 formulated with Flt3L-Fc. Draining lymph nodes and TA muscle were harvested 24 h following each vaccination and dendritic cell populations were analyzed by flow cytometry. Twenty four hours following the first vaccination, we observed no significant effect of Flt3L-Fc on DC populations at either tissue site (Figures 4A,B). Seven days later, mice administered STEAP1 formulated with Flt3L-Fc showed a significantly increased percentage of CD11c^+^MHCII^hi^ and CD11c^+^MHCII^lo^ dendritic cell populations at the site of administration, as well as a significant increase in the percent of CD11c^+^MHCII^hi^ DCs in the draining lymph node which displayed the activation markers CD80/CD86 (Figures 4C,D). The effect at the site of administration was lost 7 days later, however the percentage of both CD11c^+^MHCII^hi^ and CD11c^+^MHCII^lo^ populations was significantly increased at the lymph node at this time (Figures 4E,F).

**Figure 5:**
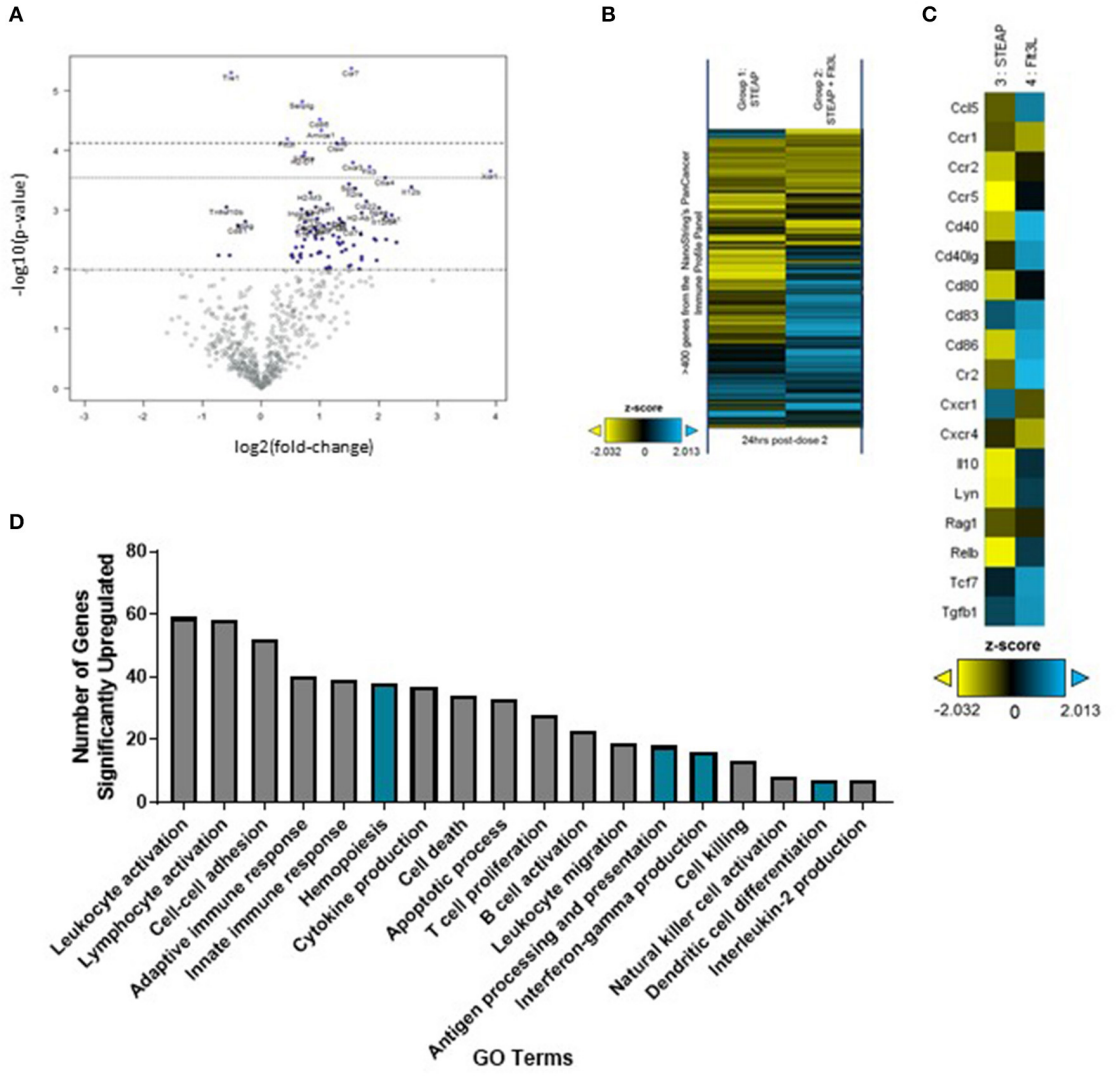
Flt3L-Fc promotes dendritic cell signaling at the site of administration. Mice were immunized weekly and the TA muscle was harvest 24 h post-dose 2. **(A)** Volcano plot analysis of differentially expressed genes at post-dose 2. **(B)** Heatmap depicting global differences in gene expression between treatment groups, following the second immunization. **(C)** Heatmap of DC function and activation genes. **(D)** Biological processes significantly upregulated by Flt3L-Fc as indicated by DAVID Gene Ontology (GO) terms. Pathways specifically associated with dendritic cells are highlighted in blue.

To further investigate the effect of Flt3L-Fc formulation at the site of administration, we extracted RNA from the TA muscle 7 days following vaccination and analyzed gene expression via the nCounter PanCancer Immune Profiling panel on the Nanostring Max Analysis System. Global differences in gene expression between mice administered STEAP1 and mice administered STEAP1 formulated with Flt3L-Fc were assessed by heat-map and volcano plot analyses (Figures 5A,B). Volcano plot analysis shows there is over 100 differentially expressed genes in TA muscles isolated from mice treated with STEAP1 plus Flt3L-Fc as compared to mice treated with STEAP1 alone (*p* < 0.01, false-discovery rate < 5%). Specifically, genes involved in DC function and/or migration such as CD86 and ITGAE were significantly upregulated (Figure 5A). Specific analysis of genes involved in DC activation and function showed that when Flt3L-Fc is formulated with STEAP1 there is a significant increase in CCL5, CD40L, CD83, and CD86 (Figure 5C). We further analyzed this dataset using DAVID (the database for annotation, visualization, and integrated discovery), a functional annotation bioinformatics resource. Biological processes which were up-regulated when Flt3L-Fc was formulated with STEAP1 included leukocyte activation, hemopoiesis, antigen processing and presentation, IFNγ production, and dendritic cell differentiation (Figure 5D, complete list of genes included in Supplementary Table 1). Combined, FACS and gene expression data show that Flt3L-Fc formulated with STEAP1 significantly enhances the DC response STEAP1 vaccine.

### Vaccine Formulation With CD80-Fc Enhances T Cell Immune Responses to Multiple Cancer Antigens

**Figure 6:**
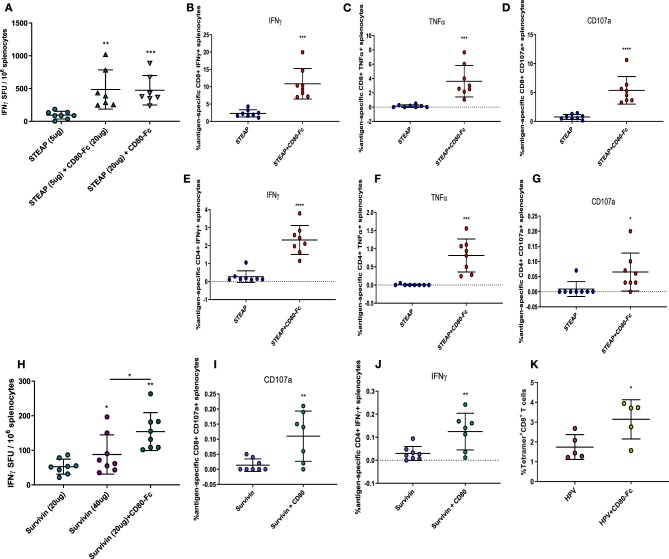
Direct targeting of T cells via CD80 ligand enhances anti-tumor antigen responses. **(A–J)** Mice were immunized biweekly according to Figure 1B and splenocytes harvested. **(B)** IFNγ ELISpot was performed to assess antigen-specific T cell responses to STEAP1. **(B–G)** Intracellular cytokine staining was done on splenocytes to characterize CD8^+^ and CD4^+^ T cell functional responses from mice immunized with STEAP1 alone or in combination with CD80-Fc. **(H)** IFNγ ELISpot was performed to assess antigen-specific T cell responses to Survivin. **(I,J)** Intracellular cytokine staining was done on splenocytes to characterize CD8^+^ and CD4^+^ T cell functional responses from mice immunized with Survivin alone or in combination with CD80-Fc. **(K)** Mice were immunized at day 0 and 21 and serially bled. PBMCs were stained with HPV E7 (H-2D^b^ RAHYNIVTF) tetramer on day 17. *N* = 8, *p < 0.05, **p < 0.01, ***p < 0.001, ****p < 0.0001.

Thus far, we have shown that indirect activation of T cells via the Flt3L adjuvant significantly boosts the antigen-specific T cell response to STEAP1. To determine the role of direct T cell engagement in our vaccination paradigm, we further investigated the effect of CD80-Fc formulation with STEAP1 on antigen-specific T cell responses. We compared two dose levels of STEAP1 (5 and 20 ug) formulated with CD80-Fc at 20 ug. As in our previous results, the addition of CD80-Fc to STEAP1 formulation at 5 ug significantly boosted the antigen-specific T cell response (Figure 6A). When STEAP1 was dosed at the maximal level, there was no further increase in antigen-specific T cell responses and both doses of STEAP1 with CD80-Fc generated antigen-specific T cell responses which were >5-fold as compared to STEAP1 alone at 5 ug. As STEAP1 alone at 20 ug compared to 5 ug resulted in a 2-fold increase only, these results indicate that this combination was not merely dose-sparing.

We proceeded to characterize the effect of CD80-Fc by specifically analyzing the T lymphocyte phenotype by flow cytometry. We performed intracellular cytokine staining on peptide-stimulated spleen cells from mice treated with STEAP1 formulated with CD80-Fc compared to STEAP1 alone. Results show that both CD8^+^ and CD4^+^ T cell populations from mice treated with STEAP1 formulated with CD80-Fc possess a significantly greater frequency of antigen-specific cell expressing IFNγ, TNFα, and CD107a compared to mice treated with STEAP1 alone (Figures 6B–G). In summary, these results indicate that CD80-Fc formulated with STEAP1 drives an increase in the quantitative and qualitative functional T cell response to the STEAP1 antigen.

Given the highly robust effect of CD80-Fc formulation with STEAP1, we examined the effect of CD80-Fc in combination with the tumor associated antigen Survivin. We compared two different dose levels of Survivin, 20 and 40 ug, chosen as the sub-optimal and maximal dose, respectively. There was a significant increase in Survivin-specific T cell responses at the 40 ug dose of Survivin compared to the 20 ug dose (Figure 6H). The addition of CD80-Fc to 20 ug of Survivin significantly enhanced the antigen-specific T cell response to levels greater than the maximal dose, indicating that the addition of CD80-Fc to Survivin vaccination was not merely dose-sparing.

We proceeded to characterize the effect of CD80-Fc by specifically analyzing the T lymphocyte phenotype by flow cytometry. We performed intracellular cytokine staining on peptide-stimulated spleen cells from mice treated with Surivivin formulated with CD80-Fc compared to Survivin alone. Results show that the CD8^+^ T cell population from mice treated with Survivin formulated with CD80-Fc possesses a significantly greater frequency of antigen-specific cells expressing CD107a compared to mice treated with Survivin alone (Figure 6I). Additionally, the CD4^+^ T cell population possessed a significantly greater frequency of antigen-specific cells expressing IFNγ compared to mice treated with Survivin alone (Figure 6J). In summary, these results indicate that CD80-Fc formulated with Survivin drives an increase in the quantitative and qualitative functional T cell response to Survivin vaccination.

Next, we examined the effect of CD80-Fc as an adjuvant to vaccination with the HPV 16 associated oncoproteins E6/E7. We measured the generation of antigen-specific T cells in the peripheral blood using a tetramer specific to the dominant epitope E7 (H-2D^b^ RAHYNIVTF). Mice were administered either HPV 16 E6/E7 pDNA alone or formulated with CD80-Fc, and RAHYNIVTF-specific T cells from the peripheral blood were quantified 2 weeks following immunization by flow cytometry. Figure 6K shows that the addition of CD80-Fc significantly increased RAHYNIVTF-specific T cells in the peripheral blood, indicating an adjuvant effect of this molecule for viral-associated cancer driving antigens. Together, these results indicate that the T cell engager and activator CD80-Fc, significantly boosts the antigen-specific T cell immune response to a diverse range of cancer-antigen targets.

### CD80-Fc in Combination With STEAP1 Reduces Tumor Burden in Mice

**Figure 7:**
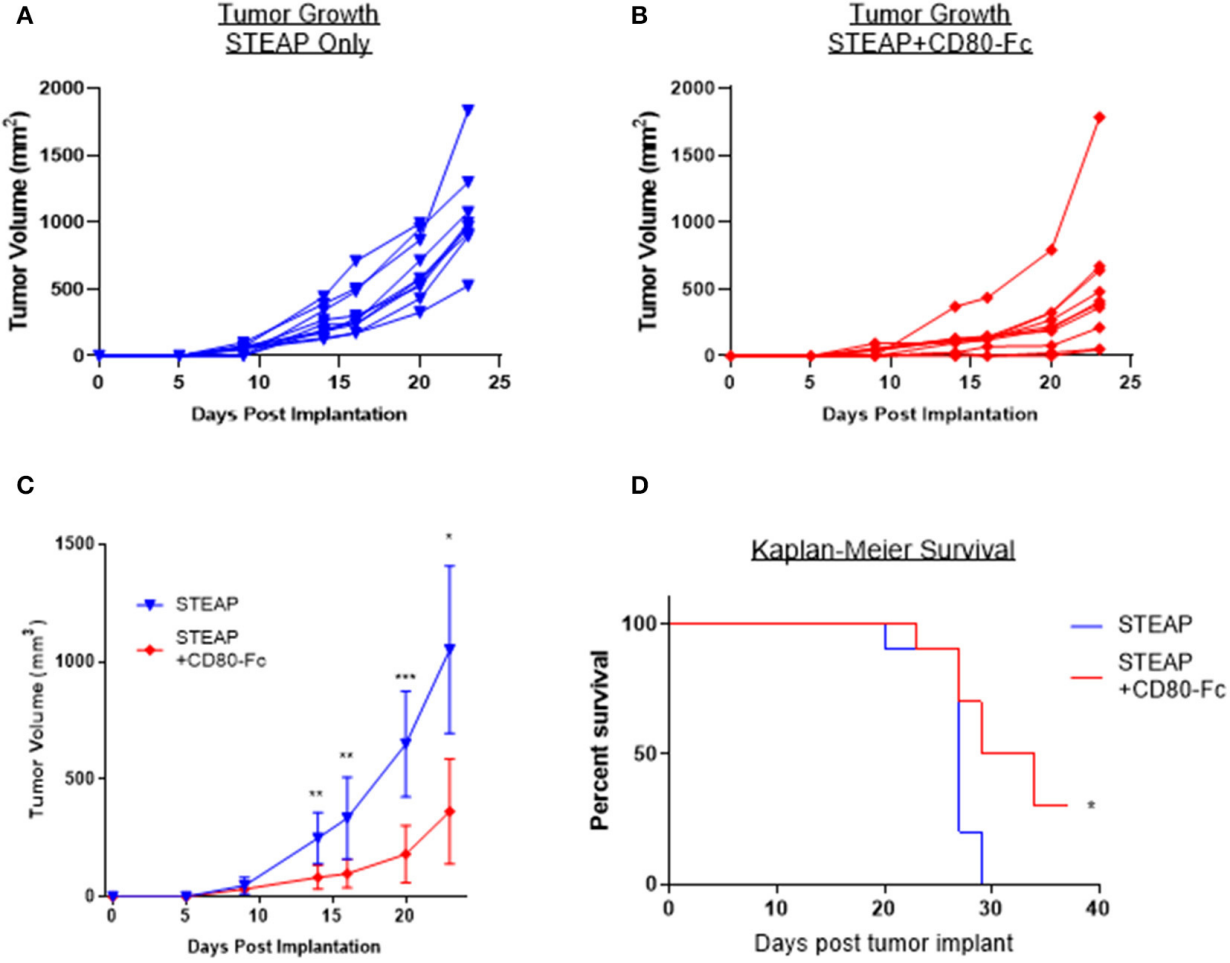
CD80-Fc in combination with STEAP1 significantly reduces tumor burden compared to STEAP1 alone. Balb/c mice were implanted subcutaneously with CT-26 tumor cells and allowed to grow to 100 mm^3^. At this time, they were separated into two groups and vaccinated with either STEAP1 alone of STEAP1 formulated with CD80-Fc. **(A,B)** Individual tumor growth curves. **(C)** Averages of A and B plotted together. **(D)** Kaplan-Meier survival curves taken at time of mouse sacrifice. *N* = 10, *p < 0.05, **p < 0.01, ***p < 0.001.

Lastly, we sought to determine the relevance of the addition of CD80-Fc with the STEAP1 vaccine in a tumor challenge model. We subcutaneously implanted CT-26 tumor cells into Balb/c mice and began vaccination when the tumors reached an average size of 100 mm^3^. Mice were vaccinated bi-weekly and tumors measured over time. Figure 7 shows that when mice were treated with STEAP1 plus CD80-Fc, there is a significant reduction in tumor growth and a significant increase in animal survival compared to mice treated with STEAP1 alone.

Collectively, the results of these data indicate that the addition of this adjuvant has a robust effect on the antigen-specific T cell response and CD80-Fc reduces tumor burden in a syngeneic mouse tumor model.

## Discussion

Robust antigen-specific T cell generation following CELLECTRA® EP enhanced delivery of pDNA vaccines has been demonstrated in several clinical trials examining a variety of antigens (12, 29). This platform has recently been expanded upon by the delivery of DNA-encoded monoclonal antibodies (dMAbs) with excellent expression kinetics *in vivo* (30–32), and a first in-human clinical trial that commenced this year (NCT03831503). The versatility of this platform is further exemplified by the recent publication describing the utility of a DNA-encoded bispecific antibody targeting Her2, which abolishes tumor growth and maintains expression in mice for several months (33). Here we further expand on the potential of our platform by showing that synthetic DNA constructs-encoding immune adjuvants can significantly enhance antigen-specific T cell responses to cancer vaccines.

In this study we designed over twenty DNA-encoded genetic adjuvants and tested their ability to enhance antigen-specific T cell responses to vaccination against two different tumor-associated antigens, STEAP1, and TERT. Although this manuscript specifically analyzed the effect of these adjuvants against these two self antigens, we hypothesize that they would also be beneficial against a personalized or neoantigen approach and future studies are underway to test this hypothesis. A more commonly used adjuvant to date is the pro-inflammatory cytokine IL-12, which has been added to vaccination trials for both infectious diseases (NCT02431767) as well as cancer vaccines (NCT03491683, NCT01440816). The utility of IL-12 stands from its ability to induce the production of IFNγ from NK and cytotoxic T cells (34) responses. In this report, we show enhanced antigen specific T cell responses to SynCon® vaccination, via directly co-stimulating T cells through the addition of a plasmid encoding CD80-Fc as well as indirectly affecting T cell activation through enhanced dendritic cell recruitment and trafficking mediated by Flt3L-Fc. It will be interesting to compare these adjuvants head-to-head with IL-12, to gain further insight to the efficacy afforded by each adjuvant tested. Additionally, while the initial screen was performed with a single dose level of antigen plus adjuvant, and therefore it is possible that other adjuvants would show benefit to vaccination following a dose level optimization analysis, dose-range follow-up studies with CD80-Fc and Flt3L-Fc indicate that the initial dose chosen was indeed optimal for these two adjuvants in combination with the STEAP1 antigen.

For optimal stimulation of naïve T cells and the induction of protective immunity three signals are required: binding of the TCR to the peptide-MHC complex, the interaction of costimulatory molecules at the interface between APCs and T cells, and cytokine production (35). Here we have focused on signal 2: stimulation of the T cell. Previous studies have revealed enhancement of signal one through advancements in pDNA delivery technology resulting in increased antigen expression. Additionally, cytokines such as IL-12 have been employed to enhance the immune response via signal 3. The data from these studies reveals that the T cell stimulation requirement could be addressed both indirectly, by enhancing their engagement with DCs via Flt3L, and directly, by the specific expression of co-stimulatory molecules such as CD80.

The use of Flt3L as an adjuvant to cancer vaccines has been previously reported (25, 36), however, this is the first study to examine the effect of Flt3L formulation with the STEAP1 antigen. We show that the addition of Flt3L-Fc to STEAP1 vaccination resulted in significantly increased antigen-specific T cell responses. The addition of Flt3L-Fc also resulted in increased migration of DCs to the site of administration and increased trafficking to the draining lymph nodes. Gene expression analysis of the treated muscle showed a significant up-regulation of genes related to DC migration and activation at day 7, however there was also increased expression of several immune checkpoint proteins including CTLA4 and PSGL1. A series of previously published studies describe a significant benefit of combining checkpoint inhibition with Flt3L therapy for the benefit of anti-tumor vaccination (37–39). Thus, it is plausible that combining the Flt3L-Fc plasmid with CTLA-4 inhibition would be synergistic and future studies will examine this combination strategy. Indeed, several papers describing the adjuvant effect of Flt3L for cancer vaccines do so in the context of a combination therapy. For example, Song et al. showed in an aggressive HBc-expressing B16 melanoma model that the addition of Flt3L combined with RANTEs to an HBc prime-boost DNA vaccination regime resulted a strong antitumor response (40). In another study, when mice were treated with a DNA vaccine encoding pN-neu in combination with either two separate monocistronic plasmids encoding Flt3L and GM-CSF, or a bicistronic plasmid encoding both cytokines, authors showed moderate, and exceptional tumor control, respectively (41). Although neither of these studies examined the effect of Flt3L alone, they do warrant the further examination of Flt3L in combination with RANTES and/or GM-CSF in CELLECTRA®-enhanced pDNA delivery protocols.

The DC-based vaccine Provenge® is the first FDA-approved vaccine for cancer therapy. DC-based vaccines, in general, are a result of the realization that DCs are a critical component to the immune response necessary to effectively target cancer, and yet are possibly defective or present in inadequate numbers (42). Of the over two dozen candidate adjuvants we screened, including chemokines and cytokines with known roles in generating an active immune response, our study showed that the T cell co-stimulators 4-1BBL, OX-40L, and CD80-Fc all significantly and robustly increased the antigen-specific T cell response to both STEAP1 and Tert, and Flt3L-Fc followed closely behind. While the Phase III clinical trial showed that Provenge® extended median survival by 4.1 months for patients with metastatic hormone-refractory prostate cancer (5), DC-based vaccines require complex *ex vivo* manipulation of cells which are then administered back to the patient for treatment. In this study we show the potential to override the defective nature of DCs harnessing their co-stimulation through the addition of CD80-Fc which directly acts on the T cells themselves. In addition to CD28, CD80 also binds PD-L1 and CTLA-4 expressed on T cells. While CTLA-4 is involved in the negative regulation of T cell activation, it is possible that CD80-FC could decrease T cell function by interacting with CTLA-4. However, numerous studies performed *in vitro* suggest that soluble CD80-Fc does not suppress T cell activation through CTLA-4 or the effect is minimal (28, 43, 44). This data is consistent with the hypothesis that CTLA-4 functions primarily as a decoy receptor for preventing CD80 activation through CD28 (45). Interestingly, our gene expression data shows that the addition of Flt3L-Fc to vaccination with STEAP1 results in an increase in CTLA-4 expression. It would be interesting to explore the combined effect of Flt3L-Fc with CD80-Fc. We would expect that the addition of CD80-Fc to a Flt3L-Fc adjuvanted vaccination could help overcome any negative regulation of T effector cells by directly engaging them via CD28, resulting in an additive or synergistic boosting. We further analyzed CD80-Fc with a variety of cancer vaccines and show that it universally led to an increased antigen-specific T cell response. Therefore, CD80-Fc presents as a potentially “off-the-shelf” approach for enhancing anti-tumor DNA vaccination.

In this article, we reveal two different methods for enhancing anti-tumor T cell responses to synthetic DNA vaccines: indirectly via Flt3L and directly via CD80. Future studies will be performed to analyze the functionality of these combinations in tumor models, however, they stand as exciting combinations for cancer vaccination and warrant further attention.

## Materials and Methods

### DNA Constructs

The SynCon® TERT and HPV type-16, E6 and E7 were generated as previously described (46–48). The SynCon® STEAP1 sequence that shares 95.6% sequence identity with human native STEAP1 was generated after performing ClustalW multiple sequence alignment using 36 STEAP1 sequences collected from human and other species. To design mouse SynCon® Survivin, four mouse survivin sequences were collected from GenBank to generate a consensus sequence. In order to abolish the potential biological function of the resulting consensus mouse survivin protein, three mutations (T34A, T48A, C84A) were introduced to abolish the anti-apoptotic activity of survivin. The resulting mouse SynCon® Survivin protein shares 95.0% identity with mouse native survivin. Once the SynCon® STEAP1 and mouse SynCon® Survivin sequences were obtained, an upstream Kozak sequence and IgE leader sequence were added to the N-terminus to have a higher level of expression. Codon/RNA optimization was also performed. The synthesized SynCon® STEAP1 and mouse SynCon® Survivin genes were cloned into Inovio's expression vector pGX0001 under the control of the human cytomegalovirus immediate-early promoter, and sequence verified for further immunogenicity study. Plasmids were purified using the Charles River Laboratories Endochrome-K Kit (Fisher Scientific), all endotoxin levels were below the detectable limit.

For the adjuvants, a series of DNA encoded murine signaling molecules with known biological effects were selected for screening in the platform. In all cases, soluble versions of molecules were designed based on known protein topologies. In some cases, murine Fc-linked versions were also constructed. Comparative protein modeling using Discovery Studio 2017 (Dassault Systèmes BIOVIA, San Diego) or Bioluminate (Schrödinger, LLC, New York, NY, 2019) was used when necessary to improve decisions based on molecule truncations and fusions, and to help in understanding potential higher-order assemblies such as dimerization upon Fc linkage.

The nucleic sequences of adjuvants used in this manuscript can be found using the following Genbank Accession Numbers: CD80–Q00609, OX40-L–P43488, 4-1BBL–P41274, IL-15–P48346, Flt3L–P49772, CCL5–CAJ18523.1, CXCL10–NP_067249.1, GM-CSF–CAA26820.1, mmCCL27–Q9Z1X0, mTSLP–Q9JIE6, CCL21a–NP_035254.1, mIL-23–Q9EQ14, IL-15RA–Q60819, mCCL3, LAG3–Q61790, mGP96–P08113. Where designs are not indicated denotes proprietary modifications to existing sequences.

### Animal Immunizations

Female BALB/c mice (6–8 weeks old) were purchased from Jackson Laboratory. All mice were housed in compliance with the Institutional Animal Care and Use Committee (IACUC) at the USDA-approved, AAALAC accredited facility ACCULAB (Sorrento Valley, San Diego, CA). Mice were immunized with 5 ug of STEAP1 plasmid with or without 20 ug of the indicated adjuvant plasmid unless otherwise described in the figure legend. DNA was formulated in Saline Sodium Citrate Buffer (SSC). Mice were injected intramuscularly (IM) into the tibialis anterior (TA) muscle followed by electroporation (EP) with the CELLECTRA® 3P device (Inovio Pharmaceuticals) as previously described (47). Briefly, two 0.1-amp constant current square-wave pulses were delivered and each pule was 52 ms in length with a 1-s delay between pulses. Vaccination schedules are indicated in the respective figures.

### ELISpot

Mouse spleens were dissociated using gentleMACS C Tubes (Miltenyi Biotec) in R10 (RPMI 1640 media supplemented with 10% FBS (Seradigm), 1% Penicillin-Streptomycin, and 0.1% 2-mercaptoethanol), followed by filtration through 40 um cell strainers (Falcon) and ACK lysis (Lonza). Splenocytes were counted and 2 × 10^5^ were added to pre-coated Mouse IFN-y ELISpot^PLUS^ (ALP) plates (Mabtech) and stimulated with overlapping 15 mer peptide pools corresponding to SynCon® or native mouse STEAP1. After 18 h at 37°C, cytokine secretion was detected according to the manufacturer's instructions. Spots were counted using an ImmunoSpot CTL reader and spot-forming units (SFU) were calculated by subtracting media alone wells from stimulated wells. Concanavalin A was used as a positive control.

### ELISA

Mouse plasma was obtained by bleeding mice through heparinized capillary tubes. Tubes were spun and the top clear layer was collected. Plasma was plated on a pre-coated Mouse Flt 3 Ligand ELISA Kit (abcam) or Mouse B7-1 ELISA Kit (abcam) and run according to the instructions of the manufacturer. Plates were analyzed at 450 nm and cytokine expression was calculated from the standard curve of each assay.

### Isolation of Peripheral Blood Mononuclear Cells (PBMCs), Lymph Node, and TA Muscle Dissociation

To isolate PBMCs, blood was collected in tubes containing 4% sodium citrate (Sigma-Aldrich) and then layered over Histopaque-1083 (Sigma-Aldrich). Tubes were spun to create a gradient and cells from the buffy coat were collected and stained for flow cytometry. Lymph nodes were mechanically disrupted using a scalpel and dissociated using an enzymatic cocktail containing R10, 100 U/ml DNase (Qiagen), and 1 mg/ml Collagenase D (Sigma-Aldrich) at 37°C for 1 h. TA muscles were dissociated using the Skeletal Muscle Dissociation kit, mouse and rat (Miltenyl Biotec, USA). Single cell suspensions were filtered and stained for flow cytometry.

### Flow Cytometry

To assess DC phenotypes, cells isolated from spleens, TA muscles, or lymph nodes were surface stained with LIVE/DEAD Aqua (Invitrogen), CD3 (145-2C11; BD Biosciences), CD19 (6D5, Biolegend), CD11c (HL3, BD Biosciences), MHC class II (M5/114.15.2, BD Biosciences), CD80 (16-10A1, BD Biosciences), and CD86 (GL1, BD Biosciences). Cell suspensions were then washed and fixed in PBS containing 1% paraformaldehyde. Samples were acquired on a FACSCanto (BD Biosciences) and analyzed using FlowJo (BD Biosciences) software. Gated CD3^−^CD19^−^CD11c^+^MHC II^+/−^ DCs were quantitated and expression of either CD80 or CD86 was considered activated.

For intracellular cytokine staining, splenocytes were stimulated with STEAP peptide pools in the presence of GolgiStop^TM^ GolgiPlug^TM^ (BD Bioscience) and CD107a (1D4B; BD Biosciences). Media alone and Cell Stimulation Cocktail (eBioscience) were used as negative and positive controls, respectively. Following stimulation, cells were washed and surface stained using LIVE/DEAD Aqua (Invitrogen), CD4 (RM4-5; BD Biosciences), CD8 (53-6.7; BD Biosciences), and CD45 (30-F11; BD Biosciences). Cells were permeabilized using the Foxp3/Transcription Factor Fixation/Permeabilization kit (eBioscience), followed by intracellular staining with CD3 (145-2C11; BD Biosciences), IFN-γ (XMG1.2; Biolegend), IL-2 (JES6-5H4, Biolegend), and TNF-α (MP6-XT22; BD Biosciences). Cells were fixed in PBS containing 1% paraformaldehyde. Samples were acquired on a FACSCanto (BD Biosciences) and analyzed using FlowJo (BD Biosciences) software. For more information on flow panels see Supplementary Tables 2, 3.

For tetramer analysis, PBMCs were washed and stained with anti-CD3 (145-2C11; eBioscience), anti-CD45 (30-F11; Biolegend), anti-CD8 (53-6.7; Biolegend), anti-CD4 (RM4-5; BD Biosciences), and the iTAg tetramer H-2Db HPV16E7 tetramer (RAHYNIVTF) (MBL). Cells were then fixed and permabilized using the FoxP3/Transcription factor fixation/permeabilization kit (eBioscience). Example FACs gating strategy for all immune cell populations can found in Supplementary Figure 1.

### Nanostring

Mouse TA muscles were extracted and flash frozen in a dry ice-methanol bath. Tissue was homogenized using a Bessman Tissue Pulverizer (VWR) in liquid nitrogen. RNA was isolated from the pulverized muscle using the RNeasy Fibrous Tissue Mini Kit (Qiagen) following the manufacturer's instructions. RNA purity was measured using a NanoDrop. The nCounter PanCancer Immune Profiling Panel (NanoString Technologies) was run according to the manufacturer's instructions and data was analyzed using nSolver Software (NanoString Technologies).

### Statistical Analysis

Statistical analysis was performed using a two-tailed unpaired Student's *t* test. Error bars represent the standard deviation. All statistical analyses were done using GraphPad Prism. *p* < 0.05 was considered statistically significant.

## Data Availability Statement

All datasets generated for this study are included in the article/Supplementary Material.

## Ethics Statement

The animal study was reviewed and approved by Institutional Animal Care and Use Committee at Acculab Life Sciences, San Diego CA.

## Author Contributions

AT, KM, and EM: conceptualization. AT and EM: methodology and supervision. AT, KM, and AW: formal analysis and writing—original. AT, KM, AW, and TN: investigation. CR, JY, NC, KB, and LH: resources. AT, TS, and EM: writing—review and editing. AT and KM: visualization.

### Conflict of Interest

JY, TS, KB, and LH are employees of Inovio Pharmaceuticals and as such receive salary and benefits, including ownership of stock and stock options. AT, KM, AW, TN, NC, EM, and CR are former employees of Inovio Pharmaceuticals.

## Supplementary Material

Supplementary Figure 1 FACs gating strategies. Example gating strategies for **(A)** functional antigen-specific T cells, **(B)** tetramer-positive T cells, and **(C)** dendritic cells.

Supplementary Table 1 Specific genes Up-regulated.

Supplementary Table 2 T cell flow cytometry panel.

Supplementary Table 3 Dendritic cell flow cytometry panel.
