# Supplementary figures and images for: Adjuvant Screen Identifies Synthetic DNA-Encoding Flt3L and CD80 Immunotherapeutics as Candidates for Enhancing Anti-tumor T Cell Responses
Source: Front Immunol. 2020 Feb 25;11:327. doi: 10.3389/fimmu.2020.00327 (PMC7052369; doi:10.3389/fimmu.2020.00327)

## Slide 1
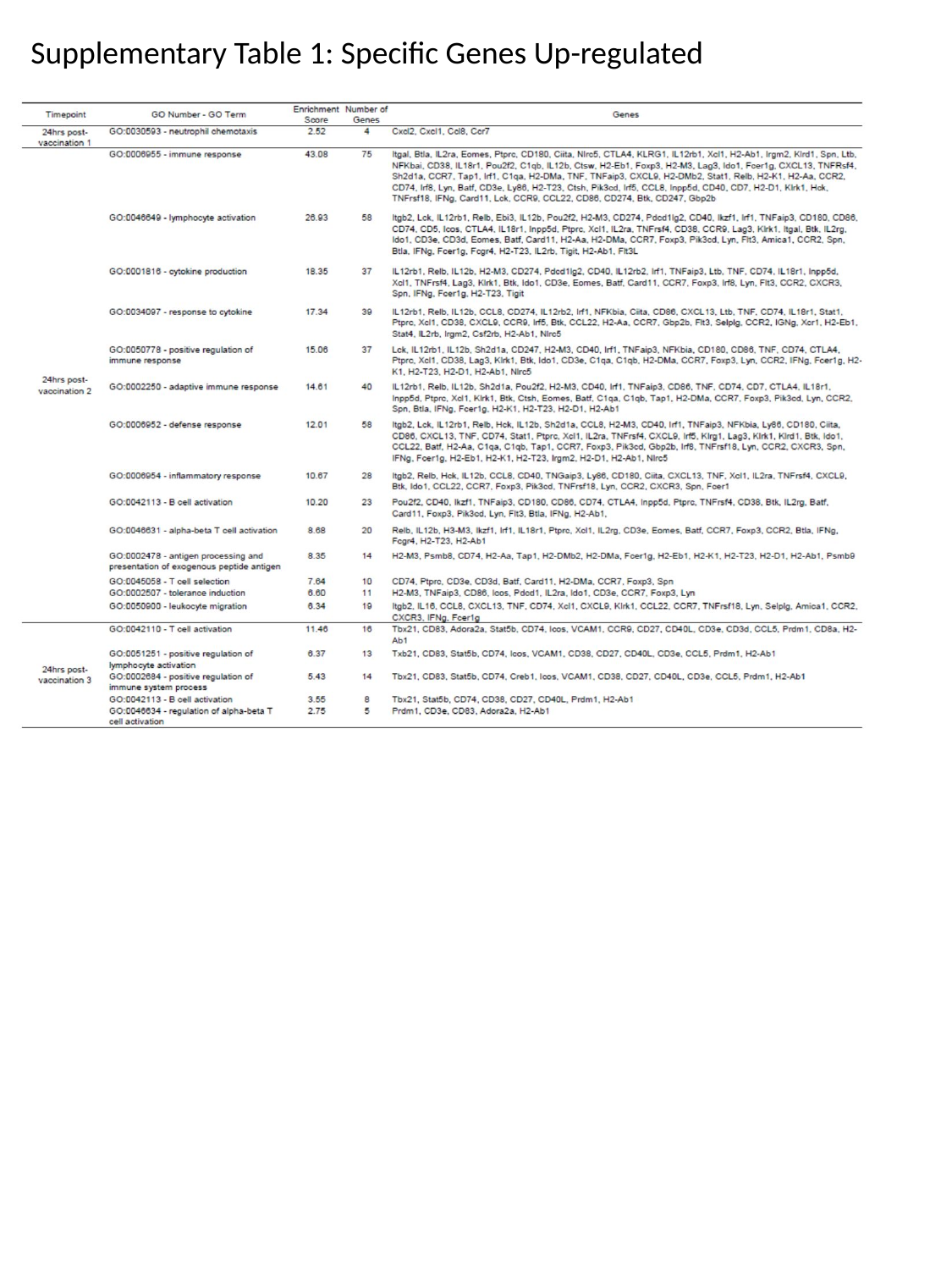

Supplementary Table 1: Specific Genes Up-regulated

Supplement: Supplementary Figure 1 — FACs gating strategies. Example gating strategies for (A) functional antigen-specific T cells, (B) tetramer-positive T cells, and (C) dendritic cells. [file Presentation_1.PPTX]

## Slide 1
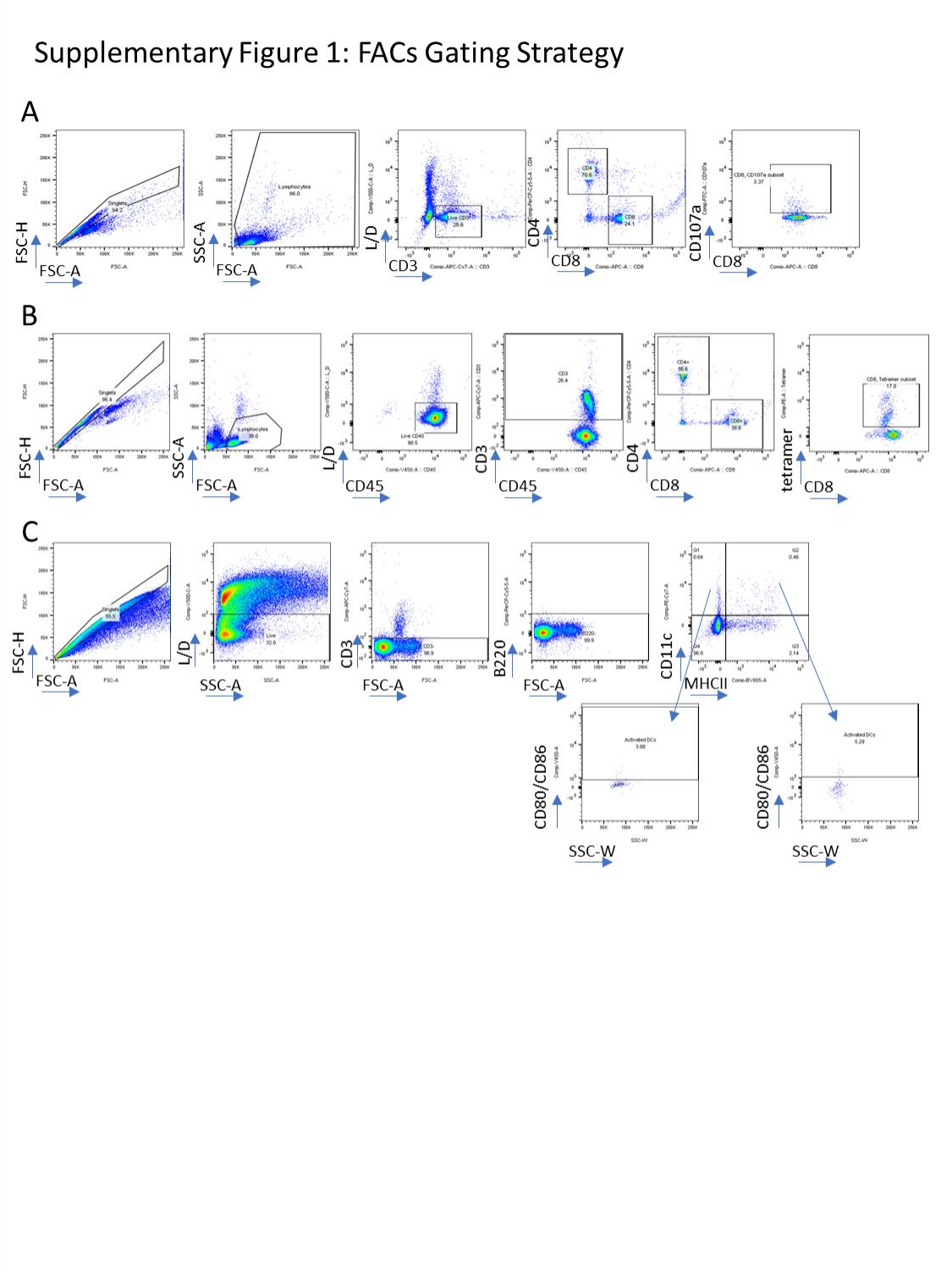

Supplement: Supplementary Table 1 — Specific genes Up-regulated. [file Presentation_2.PPTX]

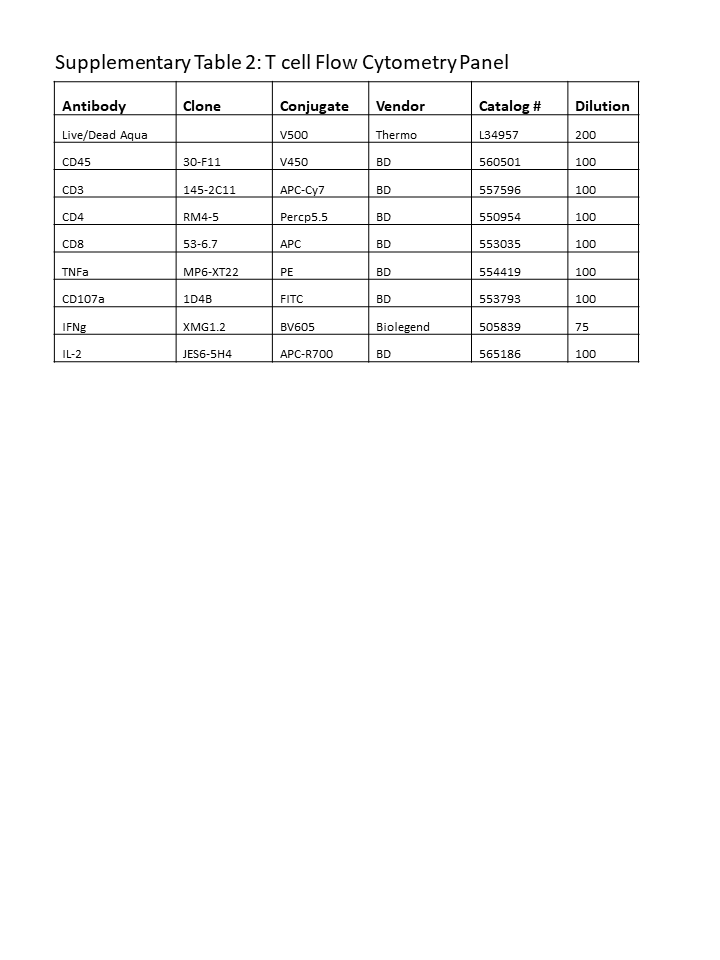

Supplement: Supplementary Table 2 — T cell flow cytometry panel. [file Image_1.TIF]

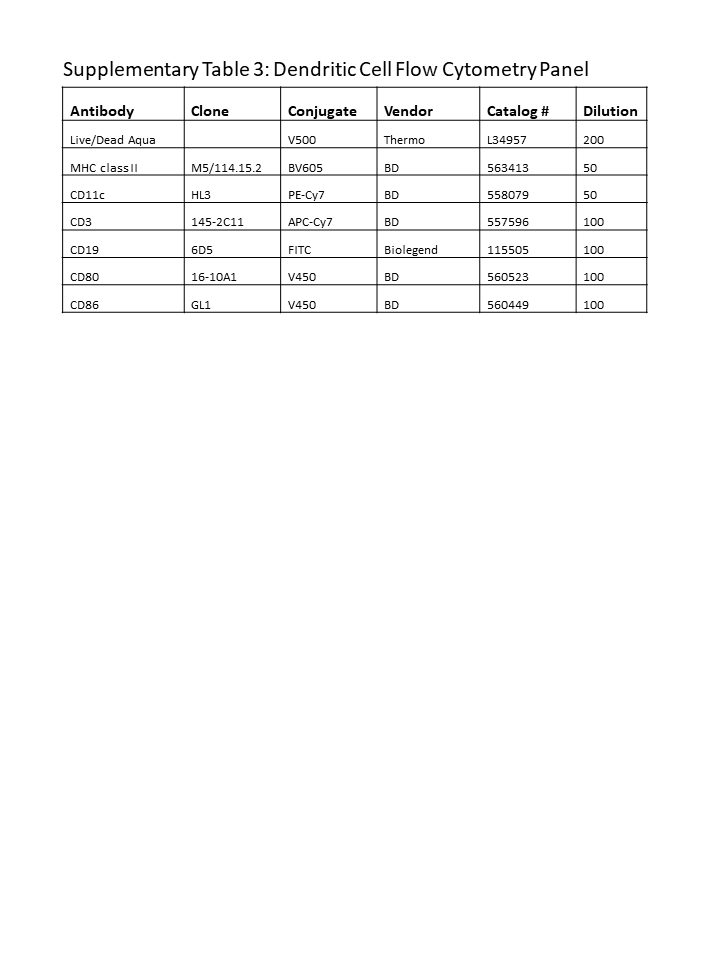

Supplement: Supplementary Table 3 — Dendritic cell flow cytometry panel. [file Image_2.TIF]
